# Analyzing the Influence of Hyper-parameters and Regularizers of Topic Modeling in Terms of Renyi Entropy

**DOI:** 10.3390/e22040394

**Published:** 2020-03-30

**Authors:** Sergei Koltcov, Vera Ignatenko, Zeyd Boukhers, Steffen Staab

**Affiliations:** 1 National Research University Higher School of Economics, Soyuza Pechatnikov Street 16, 190121 St Petersburg, Russia; vignatenko@hse.ru; 2 Institute for Web Science and Technologies, Universität Koblenz-Landau, Universitätsstrasse 1, 56070 Koblenz, Germany; boukhers@uni-koblenz.de; 3 Institute for Parallel and Distributed Systems (IPVS), Universität Stuttgart, Universitätsstraße 32, 50569 Stuttgart, Germany; Steffen.Staab@ipvs.uni-stuttgart.de; 4 Web and Internet Science Research Group, University of Southampton, University Road, Southampton SO17 1BJ, UK

**Keywords:** topic modeling, Renyi entropy, regularization

## Abstract

Topic modeling is a popular technique for clustering large collections of text documents. A variety of different types of regularization is implemented in topic modeling. In this paper, we propose a novel approach for analyzing the influence of different regularization types on results of topic modeling. Based on Renyi entropy, this approach is inspired by the concepts from statistical physics, where an inferred topical structure of a collection can be considered an information statistical system residing in a non-equilibrium state. By testing our approach on four models—Probabilistic Latent Semantic Analysis (pLSA), Additive Regularization of Topic Models (BigARTM), Latent Dirichlet Allocation (LDA) with Gibbs sampling, LDA with variational inference (VLDA)—we, first of all, show that the minimum of Renyi entropy coincides with the “true” number of topics, as determined in two labelled collections. Simultaneously, we find that Hierarchical Dirichlet Process (HDP) model as a well-known approach for topic number optimization fails to detect such optimum. Next, we demonstrate that large values of the regularization coefficient in BigARTM significantly shift the minimum of entropy from the topic number optimum, which effect is not observed for hyper-parameters in LDA with Gibbs sampling. We conclude that regularization may introduce unpredictable distortions into topic models that need further research.

## 1. Introduction

Topic modeling (TM) is one of the recent directions in statistical modeling, which is widely used in different fields such as text analysis [1], mass spectrometry [2], analysis of audio tracks [3], image analysis [4], detection and identification of nuclear isotopes [5] and many other applications. Topic models are based on a number of mathematical techniques which are related to determining hidden distributions in collections of big data. However, procedures which restore hidden distributions, possess a set of parameters such as the number of distributions in a mixture of distributions and regularization parameters. These parameters have to be set explicitly by a user of TM. In addition, the values of regularization parameters affect significantly the results of TM [6]. The problem of determining the optimal values of model parameters is complicated by the following issues. First, values of parameters can depend on the content of the analyzed dataset, correspondingly, the values of parameters can be specific for different datasets. Second, the parameters may depend on the size of the dataset. Increasing the size of a dataset makes numerical experiments on determining the optimal values of parameters to be extremely time-consuming. However, increasing the size of datasets leads to the fact that such data become comparable to mesoscopic systems and one can apply models and metrics of statistical physics to such datasets. Moreover, a part of topic models is based on different modifications of Potts model [7].

The task of TM consists of stochastic decomposition of the matrix of occurrences of words in documents ($F_{dw}$) into two matrices: (1) matrix $\Theta =(\theta_{td})$ containing the distribution of topics by documents; (2) matrix $\Phi =(\phi_{wt})$ containing the distribution of words by topics. However, stochastic matrix decomposition is defined not uniquely but with accuracy up to a non-degenerate transformation [8]. If $F_{dw}=\Phi \Theta$ is a solution then $F_{dw}=\left(\Phi R\right)\left(R^{-1}\Theta \right)$ is also a solution for all non-degenerate *R* under which $\Phi^{\prime }=\Phi R$ and $\Theta^{\prime }=R^{-1}\Theta$ are stochastic matrices. In terms of TM, ambiguity in retrieving the solution means that the algorithm starting from different initial approximations will conjugate to different points of the solution set. Namely, if running TM with the same values of parameters on the same dataset, different outputs will be obtained. It is explained by the fact that TM is an ill-posed problem [9]. The general solution to this type of problem is based on adding prior information (regularization) and modifying the sampling procedure. Furthermore, regularization can be achieved by introducing a combination of conjugate functions [1] and different types of regularization procedures [8,10]. TM parameter optimization is a significant problem that still needs an extensive research. As a partial solution, we propose an approach based on the concepts of statistical physics. Here, a collection of documents is considered an information thermodynamic system. For such a system, Renyi entropy can be introduced within the thermodynamic formalism [11] analogously to [7]. We propose an effective and universal (i.e., independent of the type of regularization) concept, based on Renyi entropy [12], for analyzing the influence of regularization on the outcome of TM. Our approach allows us to estimate optimal values of TM hyper-parameters including the number of topics and regularization parameters. Here, the optimal number of topics corresponds to the number of topics determined by encoders who label test datasets. We apply Renyi entropy approach to four topic models and two real datasets, additionally, we consider the output of hierarchical Dirichlet process model (HDP). It is important to note that the proposed approach does not apply to HDP models, which would demand its modification and, therefore, a special research. We compare the results of our approach with a standard metric in the field of machine learning, namely, log-likelihood metric and find that our method is faster and, in addition, allows to estimate the optimal number of topics while log-likelihood does not. In our work, we do not consider metrics and models related to estimation of interpretability of topic models, e.g., Kullback-Leibler divergence [13], semantic coherence [14], word intrusion [15] and others. Investigation of these metrics deserves a separate paper.

Our paper consists of the following sections. Section 2.2 describes standard metrics of quality which are used for determining parameters of topic models and considers their limitations. Section 2.1 introduces basic notations and assumptions of TM. Section 2.3 describes entropy approach where Renyi entropy is proposed as the criteria to optimize parameters and hyper-parameters in topic models. Section 3 presents the experiments carried out on two real datasets. Finally, the overall analysis of the obtained results is presented in Section 4.

## 2. Materials and Methods

### 2.1. Topic Models

Let us briefly discuss some basic ideas behind TM and introduce our notations. TM is based on the following assumptions [16]:

1. Let *D* be the number of documents in a dataset, *W* be the number of words.
2. There is a fixed number of topics (*T*) which are discussed in the dataset.
3. Datasets are regarded as sets of triples (w,d,t) from the space $\tilde{W}\times \tilde{D}\times \tilde{T}$, where $\tilde{W}$ is the set of words, $\tilde{D}$ is the set of documents, $\tilde{T}$ is the set of topics.
4. ’Bag of words’. It is supposed that the order of words in documents and the order of documents in a collection are unimportant for TM.

In TM, word probability in a document p(w|d) can be expressed as follows: $p\left(w|d\right)=\sum_{t=1}^{T}\phi_{wt}\theta_{td}$, where $\phi_{wt}$ is the probability of a word *w* to occur under a topic *t*, $\theta_{td}$ is the probability of a topic *t* in a document *d*. Probabilities $\phi_{wt}$ form a matrix of distribution of words by topics $\Phi ={\left(\phi_{wt}\right)}_{w=1,\ldots ,W;t=1,\ldots ,T}$ and probabilities $\theta_{td}$ form a matrix of distribution of topics by documents $\Theta ={\left(\theta_{td}\right)}_{t=1,\ldots ,T;d=1,\ldots ,D}$. Different types of topic models are related to different regularization algorithms. There are two main approaches in TM, namely: (1) Models which are based on maximum likelihood principle [1], where matrices Φ and Θ are searched by Expectation-Maximization (E-M) algorithm. (2) Models which are related to Monte Carlo methodology (Gibbs sampling) [17], where $\phi_{wt}$ and $\theta_{td}$ are searched by calculating expectation through Monte-Carlo method. Despite different mathematical approaches of these types of models, both of them produce similar topic solutions [17]. It is notable that topic models, regardless of the inference algorithm, transform the initial homogeneous word-topic distribution to heterogeneous distribution with low entropy. The flat (uniform) distribution is usually used as the initial distribution for LDA version with Gibbs sampling procedure, while random number generator is used for initialization of topic models with EM algorithm. In both cases, the initial distribution provides maximum entropy. During TM, the number of words with high probabilities changes significantly. In general, the output of topic modeling contains a relatively small subset of words with high probabilities (about several percents) while the rest words are assigned with probabilities about zero [18]. It should be noted that, according to numerical experiments, the percentage of highly probable words depends on the magnitude of hyper-parameters of the model and on the number of topics. These observations allows us to build a theoretical approach for analyzing such dependency using concepts of statistical physics. In our numerical experiments, five topic models are considered:

1. Probabilistic Latent Semantic Analysis (pLSA) [19] is a basic model with only one parameter—’number of topics’. Inference method for this model is based on E-M algorithm.
2. Latent Dirichlet Allocation model with Gibbs sampling procedure (LDA GS) [20] can be considered a regularized extension of pLSA, where regularization is based on prior Dirichlet distributions for Θ and Φ with parameters α and β correspondingly. Unlike the above pLSA, the inference in this model is based on Gibbs sampling procedure.
3. Variational Latent Dirichlet Allocation model (VLDA). This model uses variational E-M algorithm [1]. We consider the version of this model where regularization is based only on a prior Dirichlet distribution for Θ with parameter α. Selection of values of α is built in the algorithm.
4. The Additive Regularization of Topic Models (ARTM) [10] with smoothing/sparsing regularizers for matrix Φ (smooth/sparse phi) and matrix Θ (smooth/sparse theta), here termed sparse phi and sparse theta, respectively, is an alternative model to pLSA and LDA. These regularizers allow a user to obtain subsets of topics highly manifest in a small number of texts and/or words (sparsing effect), as well as subsets of topics relatively evenly distributed across all texts and words (smoothing effect). The parameter that controls the value of sparsing is a regularization coefficient termed τ. This model can be considered a regularization of pLSA, where regularization is embedded in E-M algorithm (regularized’ E-M algorithm).
5. Hierarchical Dirichlet Process model (HDP) is an alternative approach, providing the possibility to restore hidden topics without selecting the number of topics in advance [21,22]. Although this model is non-parametric, in real scenarios, users need to set some parameters, e.g., truncation on the allowed number of topics in the entire corpus. Since HDP returns the same number of topics as the top-level truncation that is set before, it is assumed that by discarding empty ones, the true number of topics can be obtained [22].

A more detailed description of pLSA, LDA GS, VLDA can be found in [7] (see supplementary material). For description of ARTM, we refer the reader to [10], and for HDP to [21].

### 2.2. Standard Metrics in the Field of Topic Modeling

To estimate the quality of topic models and to determine the values of parameters, three functions are most often employed for this purpose: (1) perplexity, (2) log-likelihood, (3) harmonic mean. The perplexity is a standard metric for estimating the model’s predictive capability on new data and can be expressed in the following way [23]: $perplexity\left(D_{test}\right)=\exp\left(-\frac{\sum_{d=1}^{M}\log p\left(d\right)}{\sum_{d=1}^{M}N_{d}}\right)=\exp\left(-\frac{\sum_{d=1}^{M}\sum_{w=1}^{W}n_{d}^{w}\log\left(\sum_{t=1}^{T}\phi_{wt}\theta_{td}\right)}{\sum_{d=1}^{M}N_{d}}\right)$, where $N_{d}$ is the number of words in document *d*, *M* is the number of test documents, $n_{d}^{w}$ is the number of times term *w* has been observed in document *d*. The lower the perplexity score is the better the parameters’ values are. Perplexity can also be presented as the exponent of Gibbs-Shannon entropy [24,25]. The use of perplexity for the selection of parameters of topic models is discussed in a number of works [1,20,26].

In work [26], the perplexity is used for determining the optimal number of topics. The authors demonstrated that the perplexity decreases monotonously by increasing the number of topics and does not assist in selecting the number of topics. Some works show another behaviour of perplexity, for example, authors of [17] demonstrate that the perplexity as a function of hyper-parameters has a notable unique minimum for LDA GS model, VLDA and LDA with collapsed variational Bayesian inference. Authors of [27] show that the perplexity as a function of the number of topics has a notable minimum for LDA GS model, and maximal values of perplexity correspond to T→1 and T→∞. In [28], it has been shown that the perplexity, used for a model with feature regularization, has clear minimum for some values of varying parameters and the maximum of perplexity corresponds to the maximum value of varying parameter. Thus, it can be noticed that different types of perplexity behaviour can be found in literature on TM without an explanation of such behaviour.

The use of perplexity has some limitations, which are reviewed in [29]. The authors demonstrated that the value of perplexity depends on the vocabulary size of the collection which was used for topic modeling. The dependence of perplexity value on type of topic model and size of vocabulary is shown in [30] as well. Thus, the comparison of topic models by means of perplexity is complicated [27,28] if models were implemented on different datasets and different languages. Therefore perplexity-based methods are not stable.

Another measure, which is often used when analyzing the results of topic modeling, is logarithm of likelihood which can be presented in the following way [23,31]:

$$\ln\left(P\left(\tilde{D}|\Phi ,\Theta \right)\right)=\sum_{d=1}^{D}\sum_{w=1}^{W}n_{d}^{w}\ln\left(\sum_{t=1}^{T}\phi_{wt}\theta_{td}\right),$$

where $n_{d}^{w}$ is frequency of word *w* in document *d*. Usually, the calculation of this value is carried out when the perplexity stops changing. The hyper-parameters and number of topics are selected when finding maximum of logarithm of likelihood [20]. Notice that logarithm of likelihood is a version of perplexity and different types of log-likelihood behaviour are shown in literature as well as for perplexity.

Harmonic mean is a metric that allows to evaluate how well the model can fit to the data. Considering LDA GS model, harmonic mean can be expressed as follows [32]: HM$\left({\left\{P\left(d|z^{\left(s\right)},\Phi \right)\right\}}_{s=1}^{S}\right)={\left(\frac{1}{S}\sum_{s}\frac{1}{P(d|z^{\left(s\right)},\Phi )}\right)}^{-1}$, where ${\left\{z^{\left(s\right)}\right\}}_{s=1}^{S}$ are *S* samples from a Gibbs sampler after a burn-in period, *d* is a document. Harmonic mean is used as an estimator of P(d|Φ,α). Despite the fact that harmonic mean method is simple and relatively computationally efficient, authors of many works express doubts about this method [15,32] as an evaluation technique in TM.

Let us mention that there are methods that aim to optimize hyper-parameters in the LDA model [31,33], however, they are based on log-likelihood maximization and do not consider the selection of hyper-parameters values combined with optimizing the number of topics. In addition, such methods were not tested for compliance with human judgements.

### 2.3. Entropy Approach for Analysis of Topic Models

The entropy approach is based on the idea that a large document collection can be considered an information system, for which Renyi entropy can be calculated in terms of the ’density of states’ and internal energy [7]. We theoretically assume and demonstrate experimentally that the optimal number of topics and the optimal values of hyper-parameters correspond to the minimum Renyi entropy. The ’density of states’ function can be expressed through the experimentally determined variables in the following way: ρ=N/(WT), where *N* is the number of words with relatively high probabilities (p>1/W). The internal energy is expressed through the sum of word probabilities in the following way:

(1) $$E=-\ln\left(\tilde{P}\right)=-\ln\left(\sum_{w,t}p\left(w|t\right)\cdot 1_{\left\{p(w|t)>1/W)\right\}}\right),$$

where $1_{\left\{\cdot \right\}}$ is an indicator function.

Thus, topic model is described by two observable parameters: (1) the sum of probabilities of highly probable words); (2) the number of highly probable words, *N*. Therefore, partition function (statistical sum) of a topic model can be expressed as $Z_{q}=\rho \cdot {\left(q\tilde{P}\right)}^{q}$, where q=1/T [34]. Correspondingly, Renyi entropy of a topic model is expressed in terms of partition function as

(2) $$S_{q}^{R}=\frac{\ln(Z_{q})}{1-q}.$$

A more detailed explanation of formulating Renyi entropy for topic models can be found in [7,34]. Application of Renyi entropy for investigation of TM results is useful due to the following reasons. Firstly, Renyi entropy determines the degree to which the results of TM are non-equilibrium, so it accounts for the contribution of the initial distribution of the topic model. Secondly, topic models can be optimized based on finding the minimum of Renyi entropy. Thirdly, when calculating Renyi entropy, one actually calculates the difference between two processes. Namely, increasing the number of topics, on the one hand, leads to decreasing Gibbs-Shannon entropy and, on the other hand, to increasing internal energy. What follows from this is the existence of an area where these two processes counterbalance each other. In this region, free energy and, correspondingly, Renyi entropy have the minimum values. Minimum of Renyi entropy corresponds to maximum of information of a topic model [7]. Hence, evaluation of the influence of hyper-parameters on the results of TM can be measured by means of Renyi entropy.

## 3. Results

### 3.1. Description of Data and Computer Experiments.

In our numerical experiments, the following datasets were tested:

- ’Lenta’ dataset (from lenta.ru news agency [35]). This dataset contains 8,630 documents with a vocabulary of 23,297 unique words in the Russian language. Each of these documents is manually assigned with a class from a set of 10 topic classes. However, some of these topics are strongly correlated with each other. Thus, the documents in this dataset can be represented by 7–10 topics.
- ’20 Newsgroups’ dataset [36]. This dataset consists of 15,404 news articles with 50,948 unique words. Each of the news items is assigned to one or more of 20 topic groups. Since some of these topics may be combined, 14–20 topics can represent the documents of this dataset [37].

In order to determine the influence of regularization on TM we investigated the models, which were discussed in Section 2.1, namely: (1) pLSA model [19]; (2) LDA GS model [20]; (3) VLDA model [1]; (4) BigARTM model [10]. Additionally, we compared the results of the Renyi entropy approach for determining the ’optimal’ number of topics with the results of HDP model. In our numerical experiments the number of topics *T* was varied in the range [2;50] in the increments of one topic. For LDA GS model, hyper-parameters α and β were varied in the range [0.1;1] in the increments of 0.1. For BigARTM model we used the following values of τ: 0.01, 0.1, 1, and 10. For each topic model and for each dataset we calculated log-likelihood and Renyi entropy.

Let us note that computational efficiency of Renyi entropy approach turned out to be much higher than that of log-likelihood. For instance, calculation of Renyi entropy for the Lenta dataset under variation of *T* in the range [2;50] in the increments of one took about 15 min, while calculation of log-likelihood for the same data took about nine hours. Such a great difference occurs because for Renyi entropy calculation it is enough to scan matrix Φ once, while for log-likelihood calculation one needs to multiply components of two large matrices (Φ and Θ). The purpose of our experiments was, firstly, to confirm that Renyi entropy allows us to determine the ’optimal’ number of topics for the above datasets and to compare the results of this approach with the results obtained by HDP model. Secondly, the purpose was to estimate the influence of hyper-parameters on results of TM and to specify which variant of regularization gives better results according to log-likelihood and Renyi entropy.

### 3.2. Optimal Number of Topics: HDP vs Renyi Entropy in LDA GS, VLDA and pLSA

To compare the results of HDP model, pLSA, VLDA and LDA GS, we calculate weights of topics for HDP model, and Renyi entropy for pLSA, VLDA and LDA GS. In this experiment, we used the software (available at https://github.com/chyikwei/bnp) which implements the online variational Bayes for the HDP proposed in work [22] and is optimized with cython. This algorithm was developed to analyze large datasets and is essentially faster than traditional algorithms [21,38].

**Figure 1:**
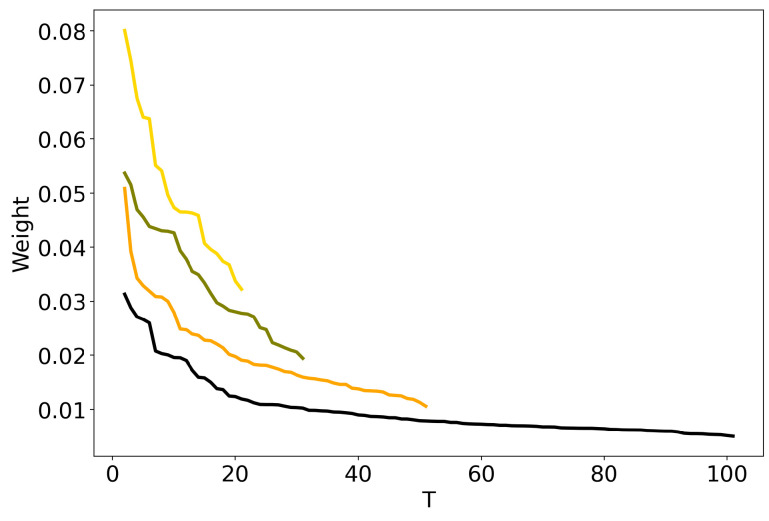
Distribution of weights over the number of topics *T* for HDP model (Lenta dataset). TLT (100)—black, TLT (50)—orange, TLT (30)–olive, TLT (20)—gold.

Figure 1 plots together the outputs of four solutions of HDP model (Lenta dataset) that differ by the values of top-level truncation parameter (TLT): 100, 50, 30, and 20. Following [39], each output is represented by a curve which sorts the weights of all inferred topics (whose number is always equal to TLT) in a descending order. The idea is to give the user an opportunity to cut off low-weight topics and to postulate that the “true” number of topics is equal to the number of high-weight topics. However, as can be seen, there is no clear threshold between high-weight and low-weight topics. The curves are monotone decreasing and do not allow to define the optimal number of topics. The same result was obtained for the 20 Newsgroups dataset. Moreover, we applied the method proposed by Wang and Blei [40] on both Russian and 20 Newsgroups corpora. This method proposes a truncation-free stochastic variational inference algorithm for HDP, which adapts the model complexity on the fly instead of requiring truncation values. For 100 runs, the method consistently inferred 28 topics on ’Lenta’ corpus and 24 topics on 20 Newsgroups corpus with default parameters. Recent progress in the inference algorithms of Bayesian nonparametric models was made in work [41] which provides promising results in terms of speed and quality. However, to the best of our knowledge, this algorithm was only applied to the tasks of image categorization but not topic modeling so far.

**Figure 2:**
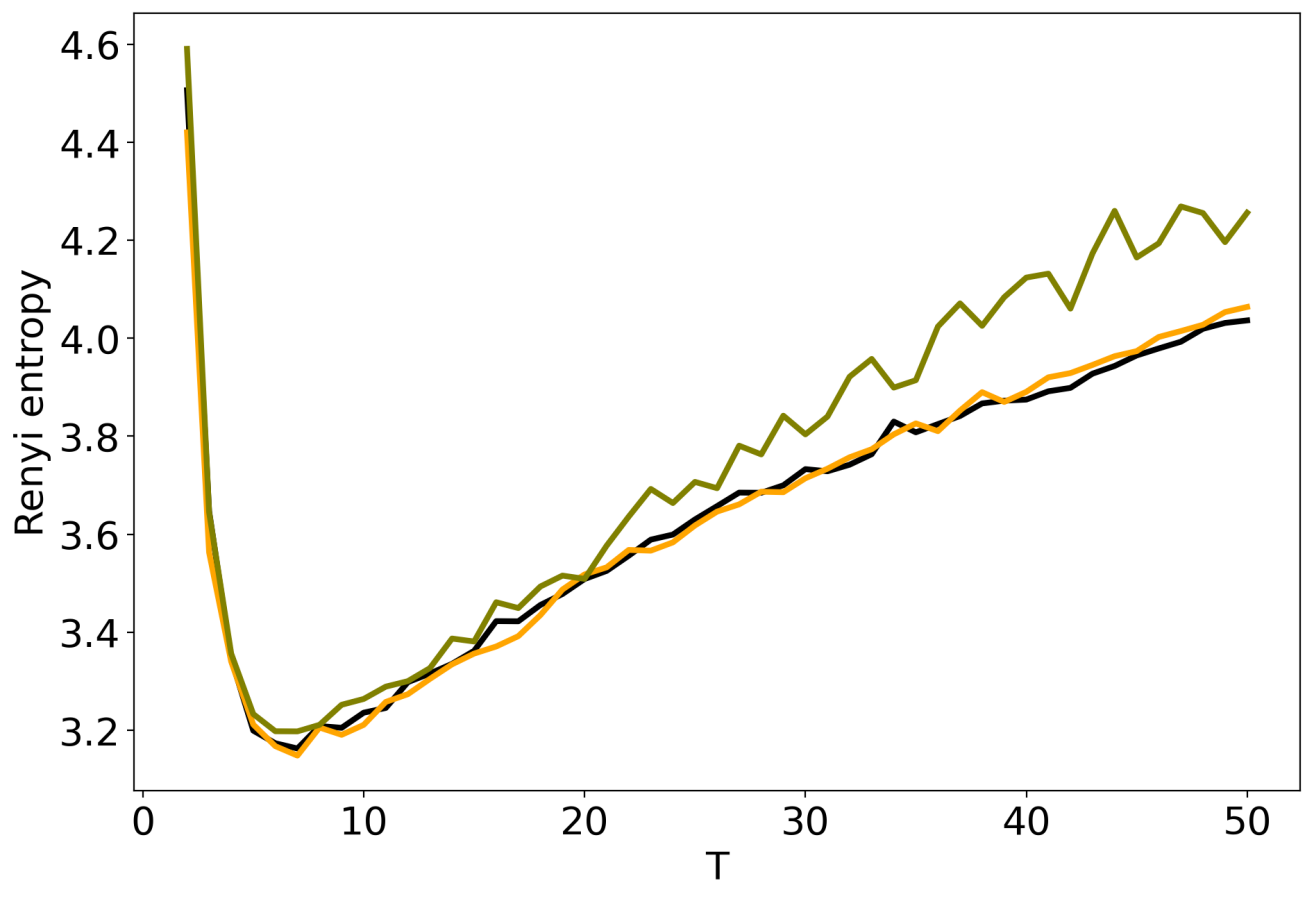
Distribution of Renyi entropy over the number of topics *T* (Lenta dataset). pLSA—black, LDA GS (β=0.1, α=0.1)–orange, VLDA—olive.

Figure 2 demonstrates Renyi entropy curves calculated according to (2) for three topic models (pLSA, LDA GS with β=0.1, α=0.5 and VLDA). For VLDA model, the number of topics was varied while the hyper-parameter α was selected automatically during the modeling. One can see that all three curves have explicit minima of entropy. Moreover, entropy curves are very similar and the locations of minima are almost identical, namely, 7–8 topics. We obtain analogous results for the 20 Newsgorups dataset. Therefore, we conclude that Renyi entropy allows us to determine the ’optimal’ number of topics for LDA GS, VLDA and pLSA models and this number is close to the human mark-up.

### 3.3. Influence of Hyper-Parameters: pLSA vs LDA GS Model

**Figure 3:**
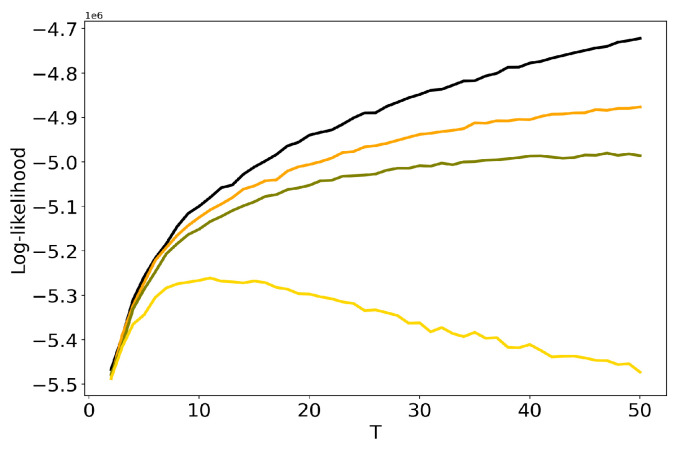
Dependence of log-likelihood on the number of topics *T* for different α and β (Lenta dataset). pLSA - black, LDA GS (α=0.1, β=0.1)—orange, LDA GS (α=0.5, β=0.1)—olive, LDA GS (α=1, β=1)—gold.

Let us discuss the influence of hyper-parameters α and β of LDA GS model on results of TM. Figure 3 demonstrates dependence of log-likelihood on the number of topics for different values of α and β (Lenta dataset). One can see that the increase in the values of hyper-parameters leads to the decrease in log-likelihood, which means that the model deteriorates as values of hyper-parameters increase. For α=β=1 we obtain the worst result. However, these curves do not allow us to determine simultaneously the optimal values of regularization parameters and the optimal number of topics. The behaviour of log-likelihood for these models on 20 Newsgroups dataset is similar to that for the Lenta dataset and, therefore, we do not provide figures.

**Figure 4:**
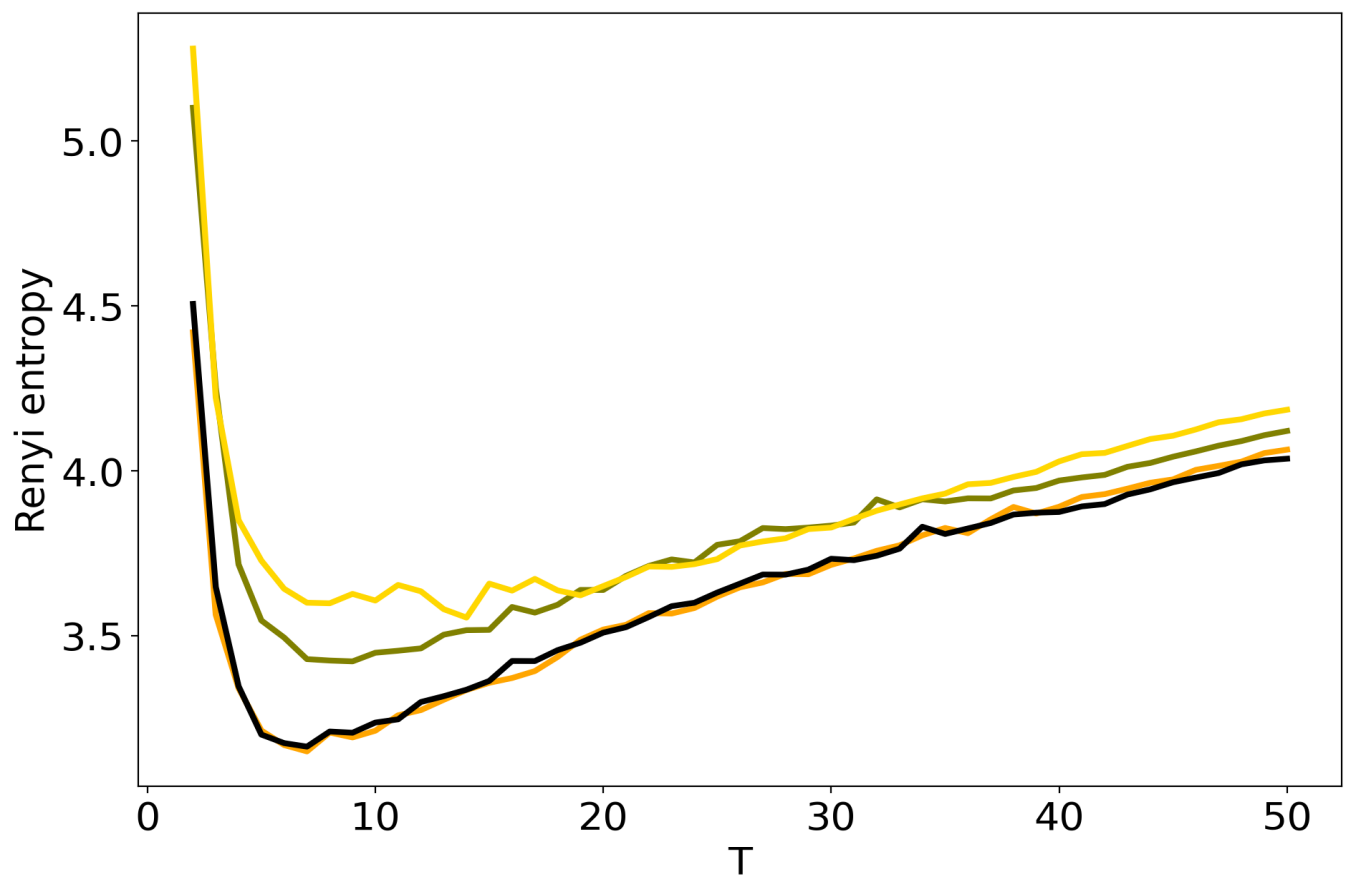
Dependence of Renyi entropy on the number of topics *T* for different α and β (Lenta dataset). pLSA—black, LDA GS (α=0.1, β=0.1)—orange, LDA GS (α=0.5, β=0.1)—olive, LDA GS (α=1, β=1)—gold.

**Figure 5:**
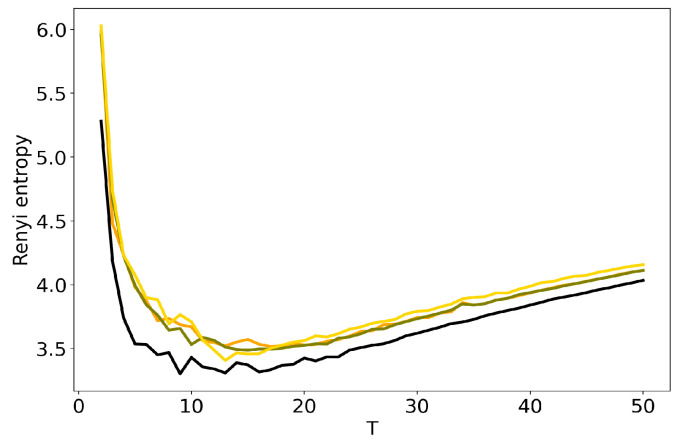
Dependence of Renyi entropy on the number of topics *T* for different α and β (20 Newsgroups dataset). pLSA—black, LDA GS (α=0.1, β=0.1)—orange, LDA GS (α=0.5, β=0.1)—olive, LDA GS (α=1, β=1)—gold.

Figure 4 and Figure 5 plot the curves of Renyi entropy for pLSA and LDA GS with different values of hyper-parameters. One can see that the increase in the values of hyper-parameters lifts the entire entropy curve, i.e., entropy increases on average. According to the entropy approach, the best model is the model with minimum entropy. It follows that the optimal models among the considered ones are pLSA and LDA GS with α=0.1, β=0.1. Notice that minima of these optimal models coincide. Numerical experiments demonstrate that minimal values of Renyi entropy for Lenta dataset are obtained with the following combinations of model parameters: (1) T=7, β=0.1, α=0.1; (2) T=9, β=0.1, α=0.5; (3) T=14, β=1, α=1. Analogously, for 20 Newsgroups dataset, the minima of Renyi entropy correspond to the following combinations of parameters: (1) T=17, β=0.1, α=0.1; (2) T=15, β=0.1, α=0.5; (3) T=13, β=1, α=1. Instability of TM leads to the fact that entropy minimum can be determined only with the accuracy up to ±3 topics [7]. Therefore, it makes more sense not to determine the exact minimum but to search for the location of a trough. Let us notice that values α=1, β=1 lead not only to the growth of the entropy values on average but also to the horizontal shift of the minimum. One can conclude that the optimal values of hyper-parameters for LDA GS model with respect to Renyi entropy are α=0.1, β=0.1. It follows that Renyi entropy approach allows us to determine both the optimal values of hyper-parameters and the optimal number of topics, while log-likelihood metric allows us to determine the optimal values of hyper-parameters only.

### 3.4. Influence of Regularization Coefficients: BigARTM vs pLSA

**Figure 6:**
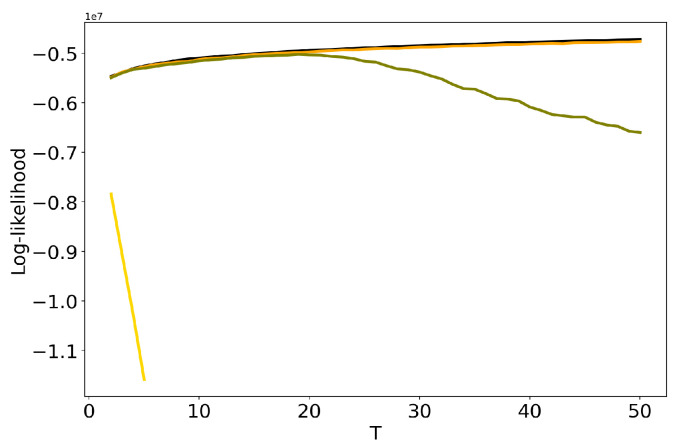
Dependence of log-likelihood on the number of topics *T* for different sparse phis (Lenta dataset): 1. pLSA—black. 2. BigARTM sparse phi (τ=0.01)—orange. 3. BigARTM sparse phi (τ=0.1)—olive. 4. BigARTM sparse phi (τ=1)—gold.

**Figure 7:**
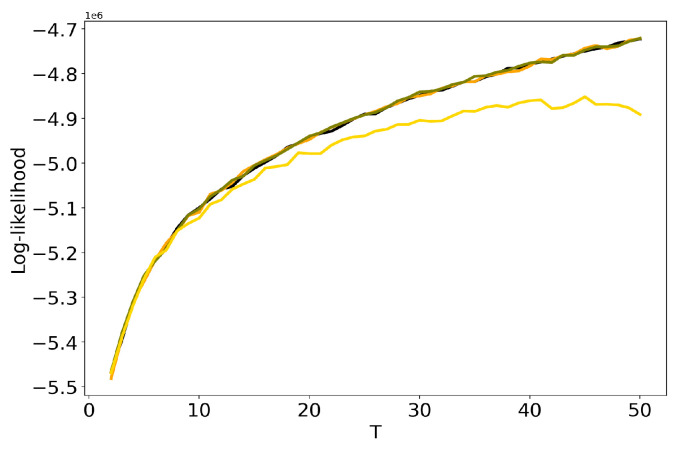
Dependence of log-likelihood on the number of topics *T* for different sparse thetas (Lenta dataset): 1. pLSA—black. 2. BigARTM sparse theta (τ=0.01)—orange. 3. BigARTM sparse theta (τ=0.1)—yellow. 4. BigARTM sparse theta (τ=1)—gold.

**Figure 8:**
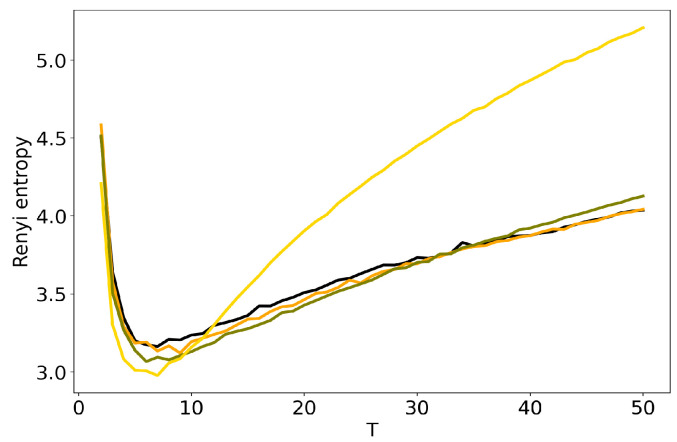
Dependence of Renyi entropy on the number of topics *T* for different sparse phis (Lenta dataset): 1. pLSA—black. 2. BigARTM sparse phi (τ=0.01)—orange. 3. BigARTM sparse phi (τ=0.1)—olive. 4. BigARTM sparse phi (τ=1)—gold.

**Figure 9:**
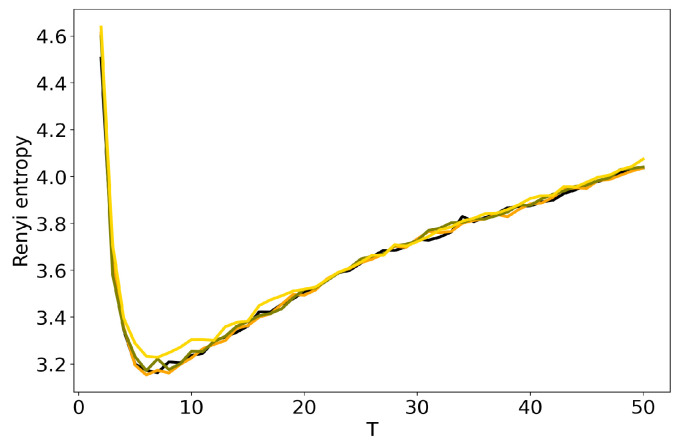
Dependence of Renyi entropy on the number of topics *T* for different sparse thetas (Lenta dataset): 1. pLSA—black. 2. BigARTM sparse theta (τ=0.01)—orange. 3. BigARTM sparse theta (τ=0.1)—olive. 4. BigARTM sparse theta (τ=1)—gold.

We further discuss the influence of regularization parameters of BigARTM model on the results of TM. Here we consider sparsing regularizers for matrix Φ (sparse phi) and matrix Θ (sparse theta), where τ is regularization coefficient. Figure 6 and Figure 7 show the behavior of log-likelihood under variation of the number of topics for different values of regularization coefficients. Both figures show that the increase in regularization coefficient impairs the model. The same result is obtained for the 20 Newsgroups dataset. Let us note that the curve of log-likelihood does not allow us to understand what happens with TM if one changes regularization coefficient and the number of topics simultaneously. Figure 8 and Figure 9 plot Renyi entropy curves for BigARTM model, which was run on the Lenta dataset under variation of the number of topics for different values of regularization coefficient. One can see that the range of coefficients [0.01;0.1] gives small fluctuations in entropy minimum. In addition, these minima are located in the range [7;10] which corresponds to the human mark-up for this dataset. However, regularization coefficient τ=1 leads to significant distortion of the Renyi entropy curve, i.e., to the lift of the entire curve and to the shift of the Renyi entropy minimum. This behavior is similar to that observed in Figure 4 and Figure 5 for hyper-parameters of LDA GS.

**Figure 10:**
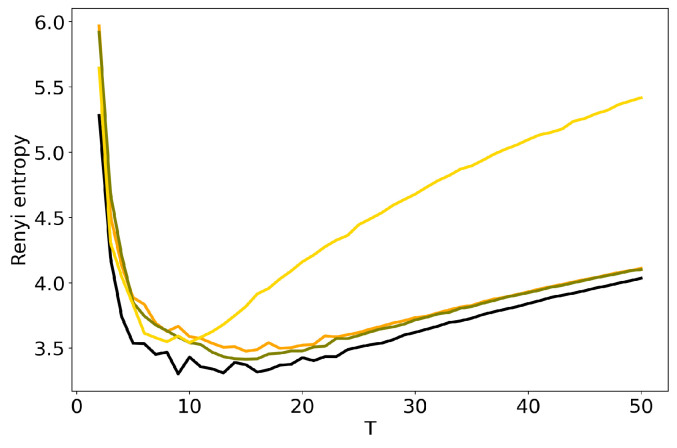
Dependence of Renyi entropy on the number of topics *T* for different sparse phis (20 Newsgroups dataset): 1. pLSA—black. 2. BigARTM sparse phi (τ=0.01)—red. 3. BigARTM sparse phi (τ=0.1)—green. 4. BigARTM sparse phi (τ=1)—blue.

**Figure 11:**
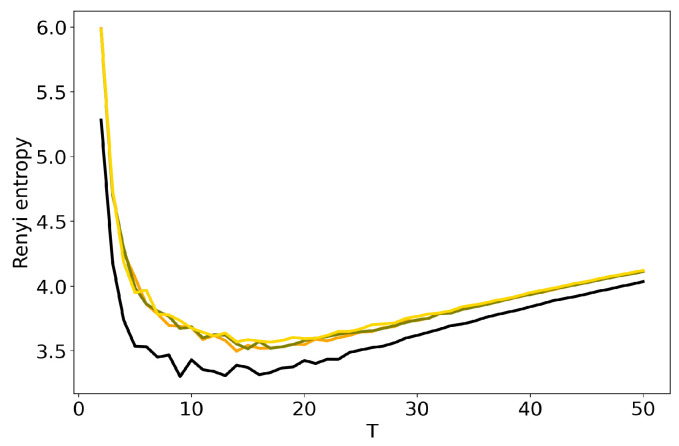
Dependence of Renyi entropy on the number of topics *T* for different sparse thetas (20 Newsgroups dataset): 1. pLSA—black. 2. BigARTM sparse theta (τ=0.01)—red. 3. BigARTM sparse theta (τ=0.1)–green. 4. BigARTM sparse theta (τ=1)—blue.

Likewise, the behavior of Renyi entropy for BigARTM on the 20 Newsgroups dataset (Figure 10 and Figure 11) is identical to that for the Lenta dataset: the curve gets distorted when τ=1. The minimum of Renyi entropy correposnds to T=10 for τ=1 in Figure 10. Additionally, in both datasets the distortion introduced by regularizing Φ is visibly larger than the effect of Θ. Our experiments show the existence of a trade-off between model quality as determined by Renyi entropy, and regularization that allows to obtain, e.g., sparse or smooth topics. In BigARTM, the smallest distortions are observed with the smallest τ which yields solutions close to the entirely unregularized model—pLSA. A similar result was obtained in [42], where pLSA was shown to perform better than any regularized BigARTM model, except the one with a dictionary-based regularizer.

## 4. Discussion

We have proposed a method based on Renyi entropy for estimating the influence of model hyper-parameters and of regularization on the results of TM. This method was tested on pLSA, LDA GS, VLDA and BigARTM models. We demonstrated that higher levels of regularization and higher values of hyper-parameters lead to lower log-likelihood and higher entropy which is a clear sign of model deterioration. They also shift the minimum of Renyi entropy away from the optimal number of topics as determined by human mark-up. However, since both metrics indicate the highest model quality there where the values of α, β and τ are low, Renyi entropy (unlike log-likelihood) may be used not only for finding the optima of those values, but also for finding an optimal number of topics, since it is in the range of low α, β and τ that Renyi entropy performs most accurately. In addition, calculation of Renyi entropy is simpler and faster than calculation of log-likelihood. Meanwhile, HDP does not provide clear thresholds to select the optimal number of topics. We conclude that Renyi entropy can be effectively used for estimating the influence of regularization coefficients and hyper-parameters on the results of TM, determining the optimal number of topics and estimating the effect of distortion under the condition of simultaneous change of multiple model parameters.

However, our work has some limitations. First, we test our approach only on two datasets in European languages. We would like to mention that these datasets were selected since they have manual markup, therefore, they can be used as ’gold standard’ datasets for testing. It would be useful to test this approach on other datasets in different languages even if they are not marked up. Second, our approach does not take into account the quality of topic solutions in the sense of semantic stability. However, it is known that regularization may lead to an increase in the stability of TM [43] that is essential for end-users of TM. This observation may lead to further development of the model parameter selection principle and deserves a separate paper.

## References

[1] Blei D.M., Ng A.Y., Jordan M.I. (2003). Latent Dirichlet Allocation. J. Mach. Learn. Res..

[2] Chernyavsky I., Alexandrov T., Maass P., Nikolenko S.I. (2012). A Two-Step Soft Segmentation Procedure for MALDI Imaging Mass Spectrometry Data. Ger. Conf. Bioinform..

[3] Yang P.H.W.L.W.J.Z. (2014). Latent topic model for audio retrieval. Pattern Recognit..

[4] Geman S., Geman D. (1984). Stochastic Relaxation, Gibbs Distributions, and the Bayesian Restoration of Images. IEEE Trans. Pattern Anal. Mach. Intell..

[5] Nelson C., Pottenger W.M., Keiler H., Grinberg N. Nuclear detection using Higher-Order topic modeling. Proceedings of the 2012 IEEE Conference on Technologies for Homeland Security (HST).

[6] George C.P., Doss H. (2017). Principled Selection of Hyperparameters in the Latent Dirichlet Allocation Model. J. Mach. Learn. Res..

[7] Koltcov S. (2018). Application of Rényi and Tsallis entropies to topic modeling optimization. Phys. A Stat. Mech. Its Appl..

[8] Vorontsov K.V. (2014). Additive regularization for topic models of text collections. Dokl. Math..

[9] Tikhonov A.N., Arsenin V.Y. (1977). Solutions of Ill-Posed Problems.

[10] Vorontsov K., Potapenko A. (2014). Tutorial on Probabilistic Topic Modeling: Additive Regularization for Stochastic Matrix Factorization. Analysis of Images, Social Networks and Texts.

[11] Rose K., Gurewitz E., Fox G.C. (1990). Statistical mechanics and phase transitions in clustering. Phys. Rev. Lett..

[12] Rényi A. (1970). Probability Theory.

[13] Steyvers M., Griffiths T. (2007). Probabilistic Topic Models.

[14] Mimno D., Wallach H.M., Talley E., Leenders M., McCallum A. Optimizing Semantic Coherence in Topic Models. Proceedings of the Conference on Empirical Methods in Natural Language Processing; Association for Computational Linguistics.

[15] Chang J., Boyd-Graber J., Gerrish S., Wang C., Blei D.M. Reading Tea Leaves: How Humans Interpret Topic Models. Proceedings of the 22nd International Conference on Neural Information Processing Systems.

[16] Hofmann T. Probabilistic Latent Semantic Indexing. Proceedings of the 22nd Annual International ACM SIGIR Conference on Research and Development in Information Retrieval.

[17] Asuncion A., Welling M., Smyth P., Teh Y.W. On Smoothing and Inference for Topic Models. Proceedings of the Twenty-Fifth Conference on Uncertainty in Artificial Intelligence.

[18] Koltcov S., Koltsova O., Nikolenko S. Latent Dirichlet Allocation: Stability and Applications to Studies of User-generated Content. Proceedings of the 2014 ACM Conference on Web Science.

[19] Hofmann T. (2001). Unsupervised Learning by Probabilistic Latent Semantic Analysis. Mach. Learn..

[20] Griffiths T.L., Steyvers M. (2004). Finding scientific topics. Proc. Natl. Acad. Sci. USA.

[21] Teh Y.W., Jordan M.I., Beal M.J., Blei D.M. (2006). Hierarchical Dirichlet Processes. J. Am. Stat. Assoc..

[22] Wang C., Paisley J., Blei D. Online Variational Inference for the Hierarchical Dirichlet Process. Proceedings of the Fourteenth International Conference on Artificial Intelligence and Statistics.

[23] Heinrich G. (2004). Parameter Estimation for Text Analysis.

[24] BUGRA Entropy and Perplexity on Image and Text. http://bugra.github.io/work/notes/2014-05-16/entropy-perplexity-image-text/.

[25] Goodman J.T. (2001). A Bit of Progress in Language Modeling. Comput. Speech Lang..

[26] Newman D., Asuncion A., Smyth P., Welling M. (2009). Distributed Algorithms for Topic Models. J. Mach. Learn. Res..

[27] Zhao W., J Chen J., Perkins R., Liu Z., Ge W., Ding Y., Zou W. A heuristic approach to determine an appropriate number of topics in topic modeling. Proceedings of the 12th Annual MCBIOS Conference.

[28] Balasubramanyan R., Dalvi B., Cohen W.W. From Topic Models to Semi-supervised Learning: Biasing Mixed-Membership Models to Exploit Topic-Indicative Features in Entity Clustering. Proceedings of the European Conference on Machine Learning and Knowledge Discovery in Databases.

[29] De Waal A., Barnard E. Evaluating topic models with stability. Proceedings of the Nineteenth Annual Symposium of the Pattern Recognition Association of South Africa.

[30] Rosen-Zvi M., Chemudugunta C., Griffiths T., Smyth P., Steyvers M. (2010). Learning Author-topic Models from Text Corpora. Acm Trans. Inf. Syst..

[31] Wallach H.M., Mimno D., McCallum A. Rethinking LDA: Why Priors Matter. Proceedings of the 22nd International Conference on Neural Information Processing Systems.

[32] Wallach H.M., Murray I., Salakhutdinov R., Mimno D. Evaluation Methods for Topic Models. Proceedings of the 26th Annual International Conference on Machine Learning.

[33] Minka T. (2000). Estimating a Dirichlet Distribution. https://tminka.github.io/papers/dirichlet/minka-dirichlet.pdf.

[34] Koltcov S., Ignatenko V., Koltsova O. (2019). Estimating Topic Modeling Performance with Sharma–Mittal Entropy. Entropy.

[35] Lenta dataset. https://www.kaggle.com/yutkin/corpus-of-russian-news-articles-from-lenta.

[36] 20Newsgroups dataset. http://qwone.com/~jason/20Newsgroups/.

[37] Basu S., Davidson I., Wagstaff K. (2008). Constrained Clustering: Advances in Algorithms, Theory, and Applications.

[38] Teh Y.W., Kurihara K., Welling M. Collapsed Variational Inference for HDP. Proceedings of the 20th International Conference on Neural Information Processing Systems.

[39] Yau C.K., Porter A., Newman N., Suominen A. (2014). Clustering scientific documents with topic modeling. Scientometrics.

[40] Wang C., Blei D.M. Truncation-free online variational inference for Bayesian nonparametric models. Proceedings of the 26th International Conference on Neural Information Processing Systems, Harrahs and Harveys.

[41] (2018). Fast approximation of variational Bayes Dirichlet process mixture using the maximization–maximization algorithm. Int. J. Approx. Reason..

[42] Apishev M., Koltcov S., Koltsova O., Nikolenko S., Vorontsov K. Additive Regularization for Topic Modeling in Sociological Studies of User-Generated Texts. Proceedings of the Mexican International Conference on Artificial Intelligence.

[43] Koltsov S., Nikolenko S., Koltsova O., Filippov V., Bodrunova S. Stable Topic Modeling with Local Density Regularization. Proceedings of the International Conference on Internet Science.

